# Measurement of dijet azimuthal decorrelation in pp collisions at $\sqrt{s}=8\,\mathrm{TeV}$

**DOI:** 10.1140/epjc/s10052-016-4346-8

**Published:** 2016-09-30

**Authors:** V. Khachatryan, A. M. Sirunyan, A. Tumasyan, W. Adam, E. Asilar, T. Bergauer, J. Brandstetter, E. Brondolin, M. Dragicevic, J. Erö, M. Flechl, M. Friedl, R. Frühwirth, V. M. Ghete, C. Hartl, N. Hörmann, J. Hrubec, M. Jeitler, V. Knünz, A. König, M. Krammer, I. Krätschmer, D. Liko, T. Matsushita, I. Mikulec, D. Rabady, N. Rad, B. Rahbaran, H. Rohringer, J. Schieck, R. Schöfbeck, J. Strauss, W. Treberer-Treberspurg, W. Waltenberger, C.-E. Wulz, V. Mossolov, N. Shumeiko, J. Suarez Gonzalez, S. Alderweireldt, T. Cornelis, E. A. De Wolf, X. Janssen, A. Knutsson, J. Lauwers, S. Luyckx, M. Van De Klundert, H. Van Haevermaet, P. Van Mechelen, N. Van Remortel, A. Van Spilbeeck, S. Abu Zeid, F. Blekman, J. D’Hondt, N. Daci, I. De Bruyn, K. Deroover, N. Heracleous, J. Keaveney, S. Lowette, L. Moreels, A. Olbrechts, Q. Python, D. Strom, S. Tavernier, W. Van Doninck, P. Van Mulders, G. P. Van Onsem, I. Van Parijs, P. Barria, H. Brun, C. Caillol, B. Clerbaux, G. De Lentdecker, G. Fasanella, L. Favart, R. Goldouzian, A. Grebenyuk, G. Karapostoli, T. Lenzi, A. Léonard, T. Maerschalk, A. Marinov, L. Perniè, A. Randle-conde, T. Seva, C. Vander Velde, P. Vanlaer, R. Yonamine, F. Zenoni, F. Zhang, K. Beernaert, L. Benucci, A. Cimmino, S. Crucy, D. Dobur, A. Fagot, G. Garcia, M. Gul, J. Mccartin, A. A. Ocampo Rios, D. Poyraz, D. Ryckbosch, S. Salva, M. Sigamani, M. Tytgat, W. Van Driessche, E. Yazgan, N. Zaganidis, S. Basegmez, C. Beluffi, O. Bondu, S. Brochet, G. Bruno, A. Caudron, L. Ceard, C. Delaere, D. Favart, L. Forthomme, A. Giammanco, A. Jafari, P. Jez, M. Komm, V. Lemaitre, A. Mertens, M. Musich, C. Nuttens, L. Perrini, K. Piotrzkowski, A. Popov, L. Quertenmont, M. Selvaggi, M. Vidal Marono, N. Beliy, G. H. Hammad, W. L. Aldá Júnior, F. L. Alves, G. A. Alves, L. Brito, M. Correa Martins Junior, M. Hamer, C. Hensel, A. Moraes, M. E. Pol, P. Rebello Teles, E. Belchior Batista Das Chagas, W. Carvalho, J. Chinellato, A. Custódio, E. M. Da Costa, D. De Jesus Damiao, C. De Oliveira Martins, S. Fonseca De Souza, L. M. Huertas Guativa, H. Malbouisson, D. Matos Figueiredo, C. Mora Herrera, L. Mundim, H. Nogima, W. L. Prado Da Silva, A. Santoro, A. Sznajder, E. J. Tonelli Manganote, A. Vilela Pereira, S. Ahuja, C. A. Bernardes, A. De Souza Santos, S. Dogra, T. R. Fernandez Perez Tomei, E. M. Gregores, P. G. Mercadante, C. S. Moon, S. F. Novaes, Sandra S. Padula, D. Romero Abad, J. C. Ruiz Vargas, A. Aleksandrov, R. Hadjiiska, P. Iaydjiev, M. Rodozov, S. Stoykova, G. Sultanov, M. Vutova, A. Dimitrov, I. Glushkov, L. Litov, B. Pavlov, P. Petkov, M. Ahmad, J. G. Bian, G. M. Chen, H. S. Chen, M. Chen, T. Cheng, R. Du, C. H. Jiang, D. Leggat, R. Plestina, F. Romeo, S. M. Shaheen, A. Spiezia, J. Tao, C. Wang, Z. Wang, H. Zhang, C. Asawatangtrakuldee, Y. Ban, Q. Li, S. Liu, Y. Mao, S. J. Qian, D. Wang, Z. Xu, C. Avila, A. Cabrera, L. F. Chaparro Sierra, C. Florez, J. P. Gomez, B. Gomez Moreno, J. C. Sanabria, N. Godinovic, D. Lelas, I. Puljak, P. M. Ribeiro Cipriano, Z. Antunovic, M. Kovac, V. Brigljevic, K. Kadija, J. Luetic, S. Micanovic, L. Sudic, A. Attikis, G. Mavromanolakis, J. Mousa, C. Nicolaou, F. Ptochos, P. A. Razis, H. Rykaczewski, M. Bodlak, M. Finger, M. Finger, E. El-khateeb, T. Elkafrawy, A. Mohamed, E. Salama, B. Calpas, M. Kadastik, M. Murumaa, M. Raidal, A. Tiko, C. Veelken, P. Eerola, J. Pekkanen, M. Voutilainen, J. Härkönen, V. Karimäki, R. Kinnunen, T. Lampén, K. Lassila-Perini, S. Lehti, T. Lindén, P. Luukka, T. Peltola, J. Tuominiemi, E. Tuovinen, L. Wendland, J. Talvitie, T. Tuuva, M. Besancon, F. Couderc, M. Dejardin, D. Denegri, B. Fabbro, J. L. Faure, C. Favaro, F. Ferri, S. Ganjour, A. Givernaud, P. Gras, G. Hamel de Monchenault, P. Jarry, E. Locci, M. Machet, J. Malcles, J. Rander, A. Rosowsky, M. Titov, A. Zghiche, I. Antropov, S. Baffioni, F. Beaudette, P. Busson, L. Cadamuro, E. Chapon, C. Charlot, O. Davignon, N. Filipovic, R. Granier de Cassagnac, M. Jo, S. Lisniak, L. Mastrolorenzo, P. Miné, I. N. Naranjo, M. Nguyen, C. Ochando, G. Ortona, P. Paganini, P. Pigard, S. Regnard, R. Salerno, J. B. Sauvan, Y. Sirois, T. Strebler, Y. Yilmaz, A. Zabi, J.-L. Agram, J. Andrea, A. Aubin, D. Bloch, J.-M. Brom, M. Buttignol, E. C. Chabert, N. Chanon, C. Collard, E. Conte, X. Coubez, J.-C. Fontaine, D. Gelé, U. Goerlach, C. Goetzmann, A.-C. Le Bihan, J. A. Merlin, K. Skovpen, P. Van Hove, S. Gadrat, S. Beauceron, C. Bernet, G. Boudoul, E. Bouvier, C. A. Carrillo Montoya, R. Chierici, D. Contardo, B. Courbon, P. Depasse, H. El Mamouni, J. Fan, J. Fay, S. Gascon, M. Gouzevitch, B. Ille, F. Lagarde, I. B. Laktineh, M. Lethuillier, L. Mirabito, A. L. Pequegnot, S. Perries, J. D. Ruiz Alvarez, D. Sabes, L. Sgandurra, V. Sordini, M. Vander Donckt, P. Verdier, S. Viret, T. Toriashvili, I. Bagaturia, C. Autermann, S. Beranek, L. Feld, A. Heister, M. K. Kiesel, K. Klein, M. Lipinski, A. Ostapchuk, M. Preuten, F. Raupach, S. Schael, J. F. Schulte, T. Verlage, H. Weber, V. Zhukov, M. Ata, M. Brodski, E. Dietz-Laursonn, D. Duchardt, M. Endres, M. Erdmann, S. Erdweg, T. Esch, R. Fischer, A. Güth, T. Hebbeker, C. Heidemann, K. Hoepfner, S. Knutzen, P. Kreuzer, M. Merschmeyer, A. Meyer, P. Millet, S. Mukherjee, M. Olschewski, K. Padeken, P. Papacz, T. Pook, M. Radziej, H. Reithler, M. Rieger, F. Scheuch, L. Sonnenschein, D. Teyssier, S. Thüer, V. Cherepanov, Y. Erdogan, G. Flügge, H. Geenen, M. Geisler, F. Hoehle, B. Kargoll, T. Kress, A. Künsken, J. Lingemann, A. Nehrkorn, A. Nowack, I. M. Nugent, C. Pistone, O. Pooth, A. Stahl, M. Aldaya Martin, I. Asin, N. Bartosik, O. Behnke, U. Behrens, K. Borras, A. Burgmeier, A. Campbell, C. Contreras-Campana, F. Costanza, C. Diez Pardos, G. Dolinska, S. Dooling, T. Dorland, G. Eckerlin, D. Eckstein, T. Eichhorn, G. Flucke, E. Gallo, J. Garay Garcia, A. Geiser, A. Gizhko, P. Gunnellini, J. Hauk, M. Hempel, H. Jung, A. Kalogeropoulos, O. Karacheban, M. Kasemann, P. Katsas, J. Kieseler, C. Kleinwort, I. Korol, W. Lange, J. Leonard, K. Lipka, A. Lobanov, W. Lohmann, R. Mankel, I.-A. Melzer-Pellmann, A. B. Meyer, G. Mittag, J. Mnich, A. Mussgiller, S. Naumann-Emme, A. Nayak, E. Ntomari, H. Perrey, D. Pitzl, R. Placakyte, A. Raspereza, B. Roland, M. Ö. Sahin, P. Saxena, T. Schoerner-Sadenius, C. Seitz, S. Spannagel, N. Stefaniuk, K. D. Trippkewitz, R. Walsh, C. Wissing, V. Blobel, M. Centis Vignali, A. R. Draeger, J. Erfle, E. Garutti, K. Goebel, D. Gonzalez, M. Görner, J. Haller, M. Hoffmann, R. S. Höing, A. Junkes, R. Klanner, R. Kogler, N. Kovalchuk, T. Lapsien, T. Lenz, I. Marchesini, D. Marconi, M. Meyer, D. Nowatschin, J. Ott, F. Pantaleo, T. Peiffer, A. Perieanu, N. Pietsch, J. Poehlsen, D. Rathjens, C. Sander, C. Scharf, P. Schleper, E. Schlieckau, A. Schmidt, S. Schumann, J. Schwandt, V. Sola, H. Stadie, G. Steinbrück, F. M. Stober, H. Tholen, D. Troendle, E. Usai, L. Vanelderen, A. Vanhoefer, B. Vormwald, C. Barth, C. Baus, J. Berger, C. Böser, E. Butz, T. Chwalek, F. Colombo, W. De Boer, A. Descroix, A. Dierlamm, S. Fink, F. Frensch, R. Friese, M. Giffels, A. Gilbert, D. Haitz, F. Hartmann, S. M. Heindl, U. Husemann, I. Katkov, A. Kornmayer, P. Lobelle Pardo, B. Maier, H. Mildner, M. U. Mozer, T. Müller, Th. Müller, M. Plagge, G. Quast, K. Rabbertz, S. Röcker, F. Roscher, M. Schröder, G. Sieber, H. J. Simonis, R. Ulrich, J. Wagner-Kuhr, S. Wayand, M. Weber, T. Weiler, S. Williamson, C. Wöhrmann, R. Wolf, G. Anagnostou, G. Daskalakis, T. Geralis, V. A. Giakoumopoulou, A. Kyriakis, D. Loukas, A. Psallidas, I. Topsis-Giotis, A. Agapitos, S. Kesisoglou, A. Panagiotou, N. Saoulidou, E. Tziaferi, I. Evangelou, G. Flouris, C. Foudas, P. Kokkas, N. Loukas, N. Manthos, I. Papadopoulos, E. Paradas, J. Strologas, G. Bencze, C. Hajdu, A. Hazi, P. Hidas, D. Horvath, F. Sikler, V. Veszpremi, G. Vesztergombi, A. J. Zsigmond, N. Beni, S. Czellar, J. Karancsi, J. Molnar, Z. Szillasi, M. Bartók, A. Makovec, P. Raics, Z. L. Trocsanyi, B. Ujvari, S. Choudhury, P. Mal, K. Mandal, D. K. Sahoo, N. Sahoo, S. K. Swain, S. Bansal, S. B. Beri, V. Bhatnagar, R. Chawla, R. Gupta, U. Bhawandeep, A. K. Kalsi, A. Kaur, M. Kaur, R. Kumar, A. Mehta, M. Mittal, J. B. Singh, G. Walia, Ashok Kumar, A. Bhardwaj, B. C. Choudhary, R. B. Garg, S. Malhotra, M. Naimuddin, N. Nishu, K. Ranjan, R. Sharma, V. Sharma, S. Bhattacharya, K. Chatterjee, S. Dey, S. Dutta, N. Majumdar, A. Modak, K. Mondal, S. Mukhopadhyay, A. Roy, D. Roy, S. Roy Chowdhury, S. Sarkar, M. Sharan, A. Abdulsalam, R. Chudasama, D. Dutta, V. Jha, V. Kumar, A. K. Mohanty, L. M. Pant, P. Shukla, A. Topkar, T. Aziz, S. Banerjee, S. Bhowmik, R. M. Chatterjee, R. K. Dewanjee, S. Dugad, S. Ganguly, S. Ghosh, M. Guchait, A. Gurtu, Sa. Jain, G. Kole, S. Kumar, B. Mahakud, M. Maity, G. Majumder, K. Mazumdar, S. Mitra, G. B. Mohanty, B. Parida, T. Sarkar, N. Sur, B. Sutar, N. Wickramage, S. Chauhan, S. Dube, A. Kapoor, K. Kothekar, S. Sharma, H. Bakhshiansohi, H. Behnamian, S. M. Etesami, A. Fahim, M. Khakzad, M. Mohammadi Najafabadi, M. Naseri, S. Paktinat Mehdiabadi, F. Rezaei Hosseinabadi, B. Safarzadeh, M. Zeinali, M. Felcini, M. Grunewald, M. Abbrescia, C. Calabria, C. Caputo, A. Colaleo, D. Creanza, L. Cristella, N. De Filippis, M. De Palma, L. Fiore, G. Iaselli, G. Maggi, M. Maggi, G. Miniello, S. My, S. Nuzzo, A. Pompili, G. Pugliese, R. Radogna, A. Ranieri, G. Selvaggi, L. Silvestris, R. Venditti, G. Abbiendi, C. Battilana, D. Bonacorsi, S. Braibant-Giacomelli, L. Brigliadori, R. Campanini, P. Capiluppi, A. Castro, F. R. Cavallo, S. S. Chhibra, G. Codispoti, M. Cuffiani, G. M. Dallavalle, F. Fabbri, A. Fanfani, D. Fasanella, P. Giacomelli, C. Grandi, L. Guiducci, S. Marcellini, G. Masetti, A. Montanari, F. L. Navarria, A. Perrotta, A. M. Rossi, T. Rovelli, G. P. Siroli, N. Tosi, G. Cappello, M. Chiorboli, S. Costa, A. Di Mattia, F. Giordano, R. Potenza, A. Tricomi, C. Tuve, G. Barbagli, V. Ciulli, C. Civinini, R. D’Alessandro, E. Focardi, V. Gori, P. Lenzi, M. Meschini, S. Paoletti, G. Sguazzoni, L. Viliani, L. Benussi, S. Bianco, F. Fabbri, D. Piccolo, F. Primavera, V. Calvelli, F. Ferro, M. Lo Vetere, M. R. Monge, E. Robutti, S. Tosi, L. Brianza, M. E. Dinardo, S. Fiorendi, S. Gennai, R. Gerosa, A. Ghezzi, P. Govoni, S. Malvezzi, R. A. Manzoni, B. Marzocchi, D. Menasce, L. Moroni, M. Paganoni, D. Pedrini, S. Ragazzi, N. Redaelli, T. Tabarelli de Fatis, S. Buontempo, N. Cavallo, S. Di Guida, M. Esposito, F. Fabozzi, A. O. M. Iorio, G. Lanza, L. Lista, S. Meola, M. Merola, P. Paolucci, C. Sciacca, F. Thyssen, P. Azzi, N. Bacchetta, L. Benato, D. Bisello, A. Boletti, A. Branca, R. Carlin, P. Checchia, M. Dall’Osso, T. Dorigo, U. Dosselli, F. Gasparini, U. Gasparini, A. Gozzelino, K. Kanishchev, S. Lacaprara, M. Margoni, A. T. Meneguzzo, M. Passaseo, J. Pazzini, M. Pegoraro, N. Pozzobon, P. Ronchese, F. Simonetto, E. Torassa, M. Tosi, M. Zanetti, P. Zotto, A. Zucchetta, A. Braghieri, A. Magnani, P. Montagna, S. P. Ratti, V. Re, C. Riccardi, P. Salvini, I. Vai, P. Vitulo, L. Alunni Solestizi, G. M. Bilei, D. Ciangottini, L. Fanò, P. Lariccia, G. Mantovani, M. Menichelli, A. Saha, A. Santocchia, K. Androsov, P. Azzurri, G. Bagliesi, J. Bernardini, T. Boccali, R. Castaldi, M. A. Ciocci, R. Dell’Orso, S. Donato, G. Fedi, L. Foà, A. Giassi, M. T. Grippo, F. Ligabue, T. Lomtadze, L. Martini, A. Messineo, F. Palla, A. Rizzi, A. Savoy-Navarro, A. T. Serban, P. Spagnolo, R. Tenchini, G. Tonelli, A. Venturi, P. G. Verdini, L. Barone, F. Cavallari, G. D’imperio, D. Del Re, M. Diemoz, S. Gelli, C. Jorda, E. Longo, F. Margaroli, P. Meridiani, G. Organtini, R. Paramatti, F. Preiato, S. Rahatlou, C. Rovelli, F. Santanastasio, P. Traczyk, N. Amapane, R. Arcidiacono, S. Argiro, M. Arneodo, R. Bellan, C. Biino, N. Cartiglia, M. Costa, R. Covarelli, A. Degano, N. Demaria, L. Finco, B. Kiani, C. Mariotti, S. Maselli, E. Migliore, V. Monaco, E. Monteil, M. M. Obertino, L. Pacher, N. Pastrone, M. Pelliccioni, G. L. Pinna Angioni, F. Ravera, A. Romero, M. Ruspa, R. Sacchi, A. Solano, A. Staiano, S. Belforte, V. Candelise, M. Casarsa, F. Cossutti, G. Della Ricca, B. Gobbo, C. La Licata, M. Marone, A. Schizzi, A. Zanetti, A. Kropivnitskaya, S. K. Nam, D. H. Kim, G. N. Kim, M. S. Kim, D. J. Kong, S. Lee, Y. D. Oh, A. Sakharov, D. C. Son, J. A. Brochero Cifuentes, H. Kim, T. J. Kim, S. Song, S. Cho, S. Choi, Y. Go, D. Gyun, B. Hong, H. Kim, Y. Kim, B. Lee, K. Lee, K. S. Lee, S. Lee, J. Lim, S. K. Park, Y. Roh, H. D. Yoo, M. Choi, H. Kim, J. H. Kim, J. S. H. Lee, I. C. Park, G. Ryu, M. S. Ryu, Y. Choi, J. Goh, D. Kim, E. Kwon, J. Lee, I. Yu, V. Dudenas, A. Juodagalvis, J. Vaitkus, I. Ahmed, Z. A. Ibrahim, J. R. Komaragiri, M. A. B. Md Ali, F. Mohamad Idris, W. A. T. Wan Abdullah, M. N. Yusli, Z. Zolkapli, E. Casimiro Linares, H. Castilla-Valdez, E. De La Cruz-Burelo, I. Heredia-De La Cruz, A. Hernandez-Almada, R. Lopez-Fernandez, A. Sanchez-Hernandez, S. Carrillo Moreno, F. Vazquez Valencia, I. Pedraza, H. A. Salazar Ibarguen, A. Morelos Pineda, D. Krofcheck, P. H. Butler, A. Ahmad, M. Ahmad, Q. Hassan, H. R. Hoorani, W. A. Khan, T. Khurshid, M. Shoaib, H. Bialkowska, M. Bluj, B. Boimska, T. Frueboes, M. Górski, M. Kazana, K. Nawrocki, K. Romanowska-Rybinska, M. Szleper, P. Zalewski, G. Brona, K. Bunkowski, A. Byszuk, K. Doroba, A. Kalinowski, M. Konecki, J. Krolikowski, M. Misiura, M. Olszewski, M. Walczak, P. Bargassa, C. Beirão Da Cruz Silva, A. Di Francesco, P. Faccioli, P. G. Ferreira Parracho, M. Gallinaro, J. Hollar, N. Leonardo, L. Lloret Iglesias, F. Nguyen, J. Rodrigues Antunes, J. Seixas, O. Toldaiev, D. Vadruccio, J. Varela, P. Vischia, S. Afanasiev, P. Bunin, M. Gavrilenko, I. Golutvin, I. Gorbunov, A. Kamenev, V. Karjavin, A. Lanev, A. Malakhov, V. Matveev, P. Moisenz, V. Palichik, V. Perelygin, S. Shmatov, S. Shulha, N. Skatchkov, V. Smirnov, A. Zarubin, V. Golovtsov, Y. Ivanov, V. Kim, E. Kuznetsova, P. Levchenko, V. Murzin, V. Oreshkin, I. Smirnov, V. Sulimov, L. Uvarov, S. Vavilov, A. Vorobyev, Yu. Andreev, A. Dermenev, S. Gninenko, N. Golubev, A. Karneyeu, M. Kirsanov, N. Krasnikov, A. Pashenkov, D. Tlisov, A. Toropin, V. Epshteyn, V. Gavrilov, N. Lychkovskaya, V. Popov, I. Pozdnyakov, G. Safronov, A. Spiridonov, E. Vlasov, A. Zhokin, A. Bylinkin, M. Chadeeva, R. Chistov, M. Danilov, V. Rusinov, V. Andreev, M. Azarkin, I. Dremin, M. Kirakosyan, A. Leonidov, G. Mesyats, S. V. Rusakov, A. Baskakov, A. Belyaev, E. Boos, M. Dubinin, L. Dudko, A. Ershov, A. Gribushin, V. Klyukhin, O. Kodolova, I. Lokhtin, I. Miagkov, S. Obraztsov, S. Petrushanko, V. Savrin, A. Snigirev, I. Azhgirey, I. Bayshev, S. Bitioukov, V. Kachanov, A. Kalinin, D. Konstantinov, V. Krychkine, V. Petrov, R. Ryutin, A. Sobol, L. Tourtchanovitch, S. Troshin, N. Tyurin, A. Uzunian, A. Volkov, P. Adzic, P. Cirkovic, J. Milosevic, V. Rekovic, J. Alcaraz Maestre, E. Calvo, M. Cerrada, M. Chamizo Llatas, N. Colino, B. De La Cruz, A. Delgado Peris, A. Escalante Del Valle, C. Fernandez Bedoya, J. P. Fernández Ramos, J. Flix, M. C. Fouz, P. Garcia-Abia, O. Gonzalez Lopez, S. Goy Lopez, J. M. Hernandez, M. I. Josa, E. Navarro De Martino, A. Pérez-Calero Yzquierdo, J. Puerta Pelayo, A. Quintario Olmeda, I. Redondo, L. Romero, J. Santaolalla, M. S. Soares, C. Albajar, J. F. de Trocóniz, M. Missiroli, D. Moran, J. Cuevas, J. Fernandez Menendez, S. Folgueras, I. Gonzalez Caballero, E. Palencia Cortezon, J. M. Vizan Garcia, I. J. Cabrillo, A. Calderon, J. R. Castiñeiras De Saa, P. De Castro Manzano, M. Fernandez, J. Garcia-Ferrero, G. Gomez, A. Lopez Virto, J. Marco, R. Marco, C. Martinez Rivero, F. Matorras, J. Piedra Gomez, T. Rodrigo, A. Y. Rodríguez-Marrero, A. Ruiz-Jimeno, L. Scodellaro, N. Trevisani, I. Vila, R. Vilar Cortabitarte, D. Abbaneo, E. Auffray, G. Auzinger, M. Bachtis, P. Baillon, A. H. Ball, D. Barney, A. Benaglia, J. Bendavid, L. Benhabib, G. M. Berruti, P. Bloch, A. Bocci, A. Bonato, C. Botta, H. Breuker, T. Camporesi, R. Castello, G. Cerminara, M. D’Alfonso, D. d’Enterria, A. Dabrowski, V. Daponte, A. David, M. De Gruttola, F. De Guio, A. De Roeck, S. De Visscher, E. Di Marco, M. Dobson, M. Dordevic, B. Dorney, T. du Pree, D. Duggan, M. Dünser, N. Dupont, A. Elliott-Peisert, G. Franzoni, J. Fulcher, W. Funk, D. Gigi, K. Gill, D. Giordano, M. Girone, F. Glege, R. Guida, S. Gundacker, M. Guthoff, J. Hammer, P. Harris, J. Hegeman, V. Innocente, P. Janot, H. Kirschenmann, M. J. Kortelainen, K. Kousouris, K. Krajczar, P. Lecoq, C. Lourenço, M. T. Lucchini, N. Magini, L. Malgeri, M. Mannelli, A. Martelli, L. Masetti, F. Meijers, S. Mersi, E. Meschi, F. Moortgat, S. Morovic, M. Mulders, M. V. Nemallapudi, H. Neugebauer, S. Orfanelli, L. Orsini, L. Pape, E. Perez, M. Peruzzi, A. Petrilli, G. Petrucciani, A. Pfeiffer, M. Pierini, D. Piparo, A. Racz, T. Reis, G. Rolandi, M. Rovere, M. Ruan, H. Sakulin, C. Schäfer, C. Schwick, M. Seidel, A. Sharma, P. Silva, M. Simon, P. Sphicas, J. Steggemann, B. Stieger, M. Stoye, Y. Takahashi, D. Treille, A. Triossi, A. Tsirou, G. I. Veres, N. Wardle, H. K. Wöhri, A. Zagozdzinska, W. D. Zeuner, W. Bertl, K. Deiters, W. Erdmann, R. Horisberger, Q. Ingram, H. C. Kaestli, D. Kotlinski, U. Langenegger, T. Rohe, F. Bachmair, L. Bäni, L. Bianchini, B. Casal, G. Dissertori, M. Dittmar, M. Donegà, P. Eller, C. Grab, C. Heidegger, D. Hits, J. Hoss, G. Kasieczka, P. Lecomte, W. Lustermann, B. Mangano, M. Marionneau, P. Martinez Ruiz del Arbol, M. Masciovecchio, M. T. Meinhard, D. Meister, F. Micheli, P. Musella, F. Nessi-Tedaldi, F. Pandolfi, J. Pata, F. Pauss, L. Perrozzi, M. Quittnat, M. Rossini, M. Schönenberger, A. Starodumov, M. Takahashi, V. R. Tavolaro, K. Theofilatos, R. Wallny, T. K. Aarrestad, C. Amsler, L. Caminada, M. F. Canelli, V. Chiochia, A. De Cosa, C. Galloni, A. Hinzmann, T. Hreus, B. Kilminster, C. Lange, J. Ngadiuba, D. Pinna, G. Rauco, P. Robmann, D. Salerno, Y. Yang, M. Cardaci, K. H. Chen, T. H. Doan, Sh. Jain, R. Khurana, M. Konyushikhin, C. M. Kuo, W. Lin, Y. J. Lu, A. Pozdnyakov, S. S. Yu, Arun Kumar, P. Chang, Y. H. Chang, Y. W. Chang, Y. Chao, K. F. Chen, P. H. Chen, C. Dietz, F. Fiori, U. Grundler, W.-S. Hou, Y. Hsiung, Y. F. Liu, R.-S. Lu, M. Miñano Moya, E. Petrakou, J. f. Tsai, Y. M. Tzeng, B. Asavapibhop, K. Kovitanggoon, G. Singh, N. Srimanobhas, N. Suwonjandee, A. Adiguzel, S. Cerci, S. Damarseckin, Z. S. Demiroglu, C. Dozen, I. Dumanoglu, E. Eskut, F. H. Gecit, S. Girgis, G. Gokbulut, Y. Guler, E. Gurpinar, I. Hos, E. E. Kangal, A. Kayis Topaksu, G. Onengut, M. Ozcan, K. Ozdemir, S. Ozturk, A. Polatoz, C. Zorbilmez, B. Bilin, S. Bilmis, B. Isildak, G. Karapinar, M. Yalvac, M. Zeyrek, E. Gülmez, M. Kaya, O. Kaya, E. A. Yetkin, T. Yetkin, A. Cakir, K. Cankocak, S. Sen, F. I. Vardarlı, B. Grynyov, L. Levchuk, P. Sorokin, R. Aggleton, F. Ball, L. Beck, J. J. Brooke, E. Clement, D. Cussans, H. Flacher, J. Goldstein, M. Grimes, G. P. Heath, H. F. Heath, J. Jacob, L. Kreczko, C. Lucas, Z. Meng, D. M. Newbold, S. Paramesvaran, A. Poll, T. Sakuma, S. Seif El Nasr-storey, S. Senkin, D. Smith, V. J. Smith, K. W. Bell, A. Belyaev, C. Brew, R. M. Brown, L. Calligaris, D. Cieri, D. J. A. Cockerill, J. A. Coughlan, K. Harder, S. Harper, E. Olaiya, D. Petyt, C. H. Shepherd-Themistocleous, A. Thea, I. R. Tomalin, T. Williams, S. D. Worm, M. Baber, R. Bainbridge, O. Buchmuller, A. Bundock, D. Burton, S. Casasso, M. Citron, D. Colling, L. Corpe, P. Dauncey, G. Davies, A. De Wit, M. Della Negra, P. Dunne, A. Elwood, D. Futyan, G. Hall, G. Iles, R. Lane, R. Lucas, L. Lyons, A.-M. Magnan, S. Malik, J. Nash, A. Nikitenko, J. Pela, M. Pesaresi, D. M. Raymond, A. Richards, A. Rose, C. Seez, A. Tapper, K. Uchida, M. Vazquez Acosta, T. Virdee, S. C. Zenz, J. E. Cole, P. R. Hobson, A. Khan, P. Kyberd, D. Leslie, I. D. Reid, P. Symonds, L. Teodorescu, M. Turner, A. Borzou, K. Call, J. Dittmann, K. Hatakeyama, H. Liu, N. Pastika, O. Charaf, S. I. Cooper, C. Henderson, P. Rumerio, D. Arcaro, A. Avetisyan, T. Bose, D. Gastler, D. Rankin, C. Richardson, J. Rohlf, L. Sulak, D. Zou, J. Alimena, E. Berry, D. Cutts, A. Ferapontov, A. Garabedian, J. Hakala, U. Heintz, O. Jesus, E. Laird, G. Landsberg, Z. Mao, M. Narain, S. Piperov, S. Sagir, R. Syarif, R. Breedon, G. Breto, M. Calderon De La Barca Sanchez, S. Chauhan, M. Chertok, J. Conway, R. Conway, P. T. Cox, R. Erbacher, G. Funk, M. Gardner, W. Ko, R. Lander, C. Mclean, M. Mulhearn, D. Pellett, J. Pilot, F. Ricci-Tam, S. Shalhout, J. Smith, M. Squires, D. Stolp, M. Tripathi, S. Wilbur, R. Yohay, R. Cousins, P. Everaerts, A. Florent, J. Hauser, M. Ignatenko, D. Saltzberg, E. Takasugi, V. Valuev, M. Weber, K. Burt, R. Clare, J. Ellison, J. W. Gary, G. Hanson, J. Heilman, M. Ivova Paneva, P. Jandir, E. Kennedy, F. Lacroix, O. R. Long, M. Malberti, M. Olmedo Negrete, A. Shrinivas, H. Wei, S. Wimpenny, B. R. Yates, J. G. Branson, G. B. Cerati, S. Cittolin, R. T. D’Agnolo, M. Derdzinski, A. Holzner, R. Kelley, D. Klein, J. Letts, I. Macneill, D. Olivito, S. Padhi, M. Pieri, M. Sani, V. Sharma, S. Simon, M. Tadel, A. Vartak, S. Wasserbaech, C. Welke, F. Würthwein, A. Yagil, G. Zevi Della Porta, J. Bradmiller-Feld, C. Campagnari, A. Dishaw, V. Dutta, K. Flowers, M. Franco Sevilla, P. Geffert, C. George, F. Golf, L. Gouskos, J. Gran, J. Incandela, N. Mccoll, S. D. Mullin, J. Richman, D. Stuart, I. Suarez, C. West, J. Yoo, D. Anderson, A. Apresyan, A. Bornheim, J. Bunn, Y. Chen, J. Duarte, A. Mott, H. B. Newman, C. Pena, M. Spiropulu, J. R. Vlimant, S. Xie, R. Y. Zhu, M. B. Andrews, V. Azzolini, A. Calamba, B. Carlson, T. Ferguson, M. Paulini, J. Russ, M. Sun, H. Vogel, I. Vorobiev, J. P. Cumalat, W. T. Ford, A. Gaz, F. Jensen, A. Johnson, M. Krohn, T. Mulholland, U. Nauenberg, K. Stenson, S. R. Wagner, J. Alexander, A. Chatterjee, J. Chaves, J. Chu, S. Dittmer, N. Eggert, N. Mirman, G. Nicolas Kaufman, J. R. Patterson, A. Rinkevicius, A. Ryd, L. Skinnari, L. Soffi, W. Sun, S. M. Tan, W. D. Teo, J. Thom, J. Thompson, J. Tucker, Y. Weng, P. Wittich, S. Abdullin, M. Albrow, G. Apollinari, S. Banerjee, L. A. T. Bauerdick, A. Beretvas, J. Berryhill, P. C. Bhat, G. Bolla, K. Burkett, J. N. Butler, H. W. K. Cheung, F. Chlebana, S. Cihangir, V. D. Elvira, I. Fisk, J. Freeman, E. Gottschalk, L. Gray, D. Green, S. Grünendahl, O. Gutsche, J. Hanlon, D. Hare, R. M. Harris, S. Hasegawa, J. Hirschauer, Z. Hu, B. Jayatilaka, S. Jindariani, M. Johnson, U. Joshi, B. Klima, B. Kreis, S. Lammel, J. Linacre, D. Lincoln, R. Lipton, T. Liu, R. Lopes De Sá, J. Lykken, K. Maeshima, J. M. Marraffino, S. Maruyama, D. Mason, P. McBride, P. Merkel, S. Mrenna, S. Nahn, C. Newman-Holmes, V. O’Dell, K. Pedro, O. Prokofyev, G. Rakness, E. Sexton-Kennedy, A. Soha, W. J. Spalding, L. Spiegel, S. Stoynev, N. Strobbe, L. Taylor, S. Tkaczyk, N. V. Tran, L. Uplegger, E. W. Vaandering, C. Vernieri, M. Verzocchi, R. Vidal, M. Wang, H. A. Weber, A. Whitbeck, D. Acosta, P. Avery, P. Bortignon, D. Bourilkov, A. Brinkerhoff, A. Carnes, M. Carver, D. Curry, S. Das, R. D. Field, I. K. Furic, S. V. Gleyzer, J. Konigsberg, A. Korytov, K. Kotov, P. Ma, K. Matchev, H. Mei, P. Milenovic, G. Mitselmakher, D. Rank, R. Rossin, L. Shchutska, M. Snowball, D. Sperka, N. Terentyev, L. Thomas, J. Wang, S. Wang, J. Yelton, S. Hewamanage, S. Linn, P. Markowitz, G. Martinez, J. L. Rodriguez, A. Ackert, J. R. Adams, T. Adams, A. Askew, S. Bein, J. Bochenek, B. Diamond, J. Haas, S. Hagopian, V. Hagopian, K. F. Johnson, A. Khatiwada, H. Prosper, M. Weinberg, M. M. Baarmand, V. Bhopatkar, S. Colafranceschi, M. Hohlmann, H. Kalakhety, D. Noonan, T. Roy, F. Yumiceva, M. R. Adams, L. Apanasevich, D. Berry, R. R. Betts, I. Bucinskaite, R. Cavanaugh, O. Evdokimov, L. Gauthier, C. E. Gerber, D. J. Hofman, P. Kurt, C. O’Brien, I. D. Sandoval Gonzalez, P. Turner, N. Varelas, Z. Wu, M. Zakaria, J. Zhang, B. Bilki, W. Clarida, K. Dilsiz, S. Durgut, R. P. Gandrajula, M. Haytmyradov, V. Khristenko, J.-P. Merlo, H. Mermerkaya, A. Mestvirishvili, A. Moeller, J. Nachtman, H. Ogul, Y. Onel, F. Ozok, A. Penzo, C. Snyder, E. Tiras, J. Wetzel, K. Yi, I. Anderson, B. A. Barnett, B. Blumenfeld, N. Eminizer, D. Fehling, L. Feng, A. V. Gritsan, P. Maksimovic, M. Osherson, J. Roskes, A. Sady, U. Sarica, M. Swartz, M. Xiao, Y. Xin, C. You, P. Baringer, A. Bean, G. Benelli, C. Bruner, R. P. Kenny, D. Majumder, M. Malek, W. Mcbrayer, M. Murray, S. Sanders, R. Stringer, Q. Wang, A. Ivanov, K. Kaadze, S. Khalil, M. Makouski, Y. Maravin, A. Mohammadi, L. K. Saini, N. Skhirtladze, S. Toda, D. Lange, F. Rebassoo, D. Wright, C. Anelli, A. Baden, O. Baron, A. Belloni, B. Calvert, S. C. Eno, C. Ferraioli, J. A. Gomez, N. J. Hadley, S. Jabeen, R. G. Kellogg, T. Kolberg, J. Kunkle, Y. Lu, A. C. Mignerey, Y. H. Shin, A. Skuja, M. B. Tonjes, S. C. Tonwar, A. Apyan, R. Barbieri, A. Baty, K. Bierwagen, S. Brandt, W. Busza, I. A. Cali, Z. Demiragli, L. Di Matteo, G. Gomez Ceballos, M. Goncharov, D. Gulhan, Y. Iiyama, G. M. Innocenti, M. Klute, D. Kovalskyi, Y. S. Lai, Y.-J. Lee, A. Levin, P. D. Luckey, A. C. Marini, C. Mcginn, C. Mironov, S. Narayanan, X. Niu, C. Paus, C. Roland, G. Roland, J. Salfeld-Nebgen, G. S. F. Stephans, K. Sumorok, M. Varma, D. Velicanu, J. Veverka, J. Wang, T. W. Wang, B. Wyslouch, M. Yang, V. Zhukova, A. C. Benvenuti, B. Dahmes, A. Evans, A. Finkel, A. Gude, P. Hansen, S. Kalafut, S. C. Kao, K. Klapoetke, Y. Kubota, Z. Lesko, J. Mans, S. Nourbakhsh, N. Ruckstuhl, R. Rusack, N. Tambe, J. Turkewitz, J. G. Acosta, S. Oliveros, E. Avdeeva, R. Bartek, K. Bloom, S. Bose, D. R. Claes, A. Dominguez, C. Fangmeier, R. Gonzalez Suarez, R. Kamalieddin, D. Knowlton, I. Kravchenko, F. Meier, J. Monroy, F. Ratnikov, J. E. Siado, G. R. Snow, M. Alyari, J. Dolen, J. George, A. Godshalk, C. Harrington, I. Iashvili, J. Kaisen, A. Kharchilava, A. Kumar, S. Rappoccio, B. Roozbahani, G. Alverson, E. Barberis, D. Baumgartel, M. Chasco, A. Hortiangtham, A. Massironi, D. M. Morse, D. Nash, T. Orimoto, R. Teixeira De Lima, D. Trocino, R.-J. Wang, D. Wood, J. Zhang, S. Bhattacharya, K. A. Hahn, A. Kubik, J. F. Low, N. Mucia, N. Odell, B. Pollack, M. Schmitt, K. Sung, M. Trovato, M. Velasco, N. Dev, M. Hildreth, C. Jessop, D. J. Karmgard, N. Kellams, K. Lannon, N. Marinelli, F. Meng, C. Mueller, Y. Musienko, M. Planer, A. Reinsvold, R. Ruchti, G. Smith, S. Taroni, N. Valls, M. Wayne, M. Wolf, A. Woodard, L. Antonelli, J. Brinson, B. Bylsma, L. S. Durkin, S. Flowers, A. Hart, C. Hill, R. Hughes, W. Ji, T. Y. Ling, B. Liu, W. Luo, D. Puigh, M. Rodenburg, B. L. Winer, H. W. Wulsin, O. Driga, P. Elmer, J. Hardenbrook, P. Hebda, S. A. Koay, P. Lujan, D. Marlow, T. Medvedeva, M. Mooney, J. Olsen, C. Palmer, P. Piroué, D. Stickland, C. Tully, A. Zuranski, S. Malik, A. Barker, V. E. Barnes, D. Benedetti, D. Bortoletto, L. Gutay, M. K. Jha, M. Jones, A. W. Jung, K. Jung, A. Kumar, D. H. Miller, N. Neumeister, B. C. Radburn-Smith, X. Shi, I. Shipsey, D. Silvers, J. Sun, A. Svyatkovskiy, F. Wang, W. Xie, L. Xu, N. Parashar, J. Stupak, A. Adair, B. Akgun, Z. Chen, K. M. Ecklund, F. J. M. Geurts, M. Guilbaud, W. Li, B. Michlin, M. Northup, B. P. Padley, R. Redjimi, J. Roberts, J. Rorie, Z. Tu, J. Zabel, B. Betchart, A. Bodek, P. de Barbaro, R. Demina, Y. Eshaq, T. Ferbel, M. Galanti, A. Garcia-Bellido, J. Han, A. Harel, O. Hindrichs, A. Khukhunaishvili, K. H. Lo, G. Petrillo, P. Tan, M. Verzetti, J. P. Chou, E. Contreras-Campana, D. Ferencek, Y. Gershtein, E. Halkiadakis, M. Heindl, D. Hidas, E. Hughes, S. Kaplan, R. Kunnawalkam Elayavalli, A. Lath, K. Nash, H. Saka, S. Salur, S. Schnetzer, D. Sheffield, S. Somalwar, R. Stone, S. Thomas, P. Thomassen, M. Walker, M. Foerster, G. Riley, K. Rose, S. Spanier, K. Thapa, O. Bouhali, A. Castaneda Hernandez, A. Celik, M. Dalchenko, M. De Mattia, A. Delgado, S. Dildick, R. Eusebi, J. Gilmore, T. Huang, T. Kamon, V. Krutelyov, R. Mueller, I. Osipenkov, Y. Pakhotin, R. Patel, A. Perloff, A. Rose, A. Safonov, A. Tatarinov, K. A. Ulmer, N. Akchurin, C. Cowden, J. Damgov, C. Dragoiu, P. R. Dudero, J. Faulkner, S. Kunori, K. Lamichhane, S. W. Lee, T. Libeiro, S. Undleeb, I. Volobouev, E. Appelt, A. G. Delannoy, S. Greene, A. Gurrola, R. Janjam, W. Johns, C. Maguire, Y. Mao, A. Melo, H. Ni, P. Sheldon, S. Tuo, J. Velkovska, Q. Xu, M. W. Arenton, B. Cox, B. Francis, J. Goodell, R. Hirosky, A. Ledovskoy, H. Li, C. Lin, C. Neu, T. Sinthuprasith, X. Sun, Y. Wang, E. Wolfe, J. Wood, F. Xia, C. Clarke, R. Harr, P. E. Karchin, C. Kottachchi Kankanamge Don, P. Lamichhane, J. Sturdy, D. A. Belknap, D. Carlsmith, M. Cepeda, S. Dasu, L. Dodd, S. Duric, B. Gomber, M. Grothe, M. Herndon, A. Hervé, P. Klabbers, A. Lanaro, A. Levine, K. Long, R. Loveless, A. Mohapatra, I. Ojalvo, T. Perry, G. A. Pierro, G. Polese, T. Ruggles, T. Sarangi, A. Savin, A. Sharma, N. Smith, W. H. Smith, D. Taylor, P. Verwilligen, N. Woods, [Authorinst]The CMS Collaboration

**Affiliations:** 1 Yerevan Physics Institute, Yerevan, Armenia; 2 Institut für Hochenergiephysik der OeAW, Vienna, Austria; 3 National Centre for Particle and High Energy Physics, Minsk, Belarus; 4 Universiteit Antwerpen, Antwerp, Belgium; 5 Vrije Universiteit Brussel, Brussels, Belgium; 6 Université Libre de Bruxelles, Brussels, Belgium; 7 Ghent University, Ghent, Belgium; 8 Université Catholique de Louvain, Louvain-la-Neuve, Belgium; 9 Université de Mons, Mons, Belgium; 10 Centro Brasileiro de Pesquisas Fisicas, Rio de Janeiro, Brazil; 11 Universidade do Estado do Rio de Janeiro, Rio de Janeiro, Brazil; 12 Universidade Estadual Paulista, Universidade Federal do ABC, São Paulo, Brazil; 13 Institute for Nuclear Research and Nuclear Energy, Sofia, Bulgaria; 14 University of Sofia, Sofia, Bulgaria; 15 Institute of High Energy Physics, Beijing, China; 16 State Key Laboratory of Nuclear Physics and Technology, Peking University, Beijing, China; 17 Universidad de Los Andes, Bogotá, Colombia; 18 Faculty of Electrical Engineering, Mechanical Engineering and Naval Architecture, University of Split, Split, Croatia; 19 Faculty of Science, University of Split, Split, Croatia; 20 Institute Rudjer Boskovic, Zagreb, Croatia; 21 University of Cyprus, Nicosia, Cyprus; 22 Charles University, Prague, Czech Republic; 23 Academy of Scientific Research and Technology of the Arab Republic of Egypt, Egyptian Network of High Energy Physics, Cairo, Egypt; 24 National Institute of Chemical Physics and Biophysics, Tallinn, Estonia; 25 Department of Physics, University of Helsinki, Helsinki, Finland; 26 Helsinki Institute of Physics, Helsinki, Finland; 27 Lappeenranta University of Technology, Lappeenranta, Finland; 28 DSM/IRFU, CEA/Saclay, Gif-sur-Yvette, France; 29 Laboratoire Leprince-Ringuet, Ecole Polytechnique, IN2P3-CNRS, Palaiseau, France; 30 Institut Pluridisciplinaire Hubert Curien, Université de Strasbourg, Université de Haute Alsace Mulhouse, CNRS/IN2P3, Strasbourg, France; 31 Centre de Calcul de l’Institut National de Physique Nucleaire et de Physique des Particules CNRS/IN2P3, Villeurbanne, France; 32 Institut de Physique Nucléaire de Lyon, Université de Lyon, Université Claude Bernard Lyon 1, CNRS-IN2P3, Villeurbanne, France; 33 Georgian Technical University, Tbilisi, Georgia; 34 Tbilisi State University, Tbilisi, Georgia; 35 RWTH Aachen University, I. Physikalisches Institut, Aachen, Germany; 36 RWTH Aachen University, III. Physikalisches Institut A, Aachen, Germany; 37 RWTH Aachen University, III. Physikalisches Institut B, Aachen, Germany; 38 Deutsches Elektronen-Synchrotron, Hamburg, Germany; 39 University of Hamburg, Hamburg, Germany; 40 Institut für Experimentelle Kernphysik, Karlsruhe, Germany; 41 Institute of Nuclear and Particle Physics (INPP), NCSR Demokritos, Aghia Paraskevi, Greece; 42 National and Kapodistrian University of Athens, Athens, Greece; 43 University of Ioánnina, Ioannina, Greece; 44 Wigner Research Centre for Physics, Budapest, Hungary; 45 Institute of Nuclear Research ATOMKI, Debrecen, Hungary; 46 University of Debrecen, Debrecen, Hungary; 47 National Institute of Science Education and Research, Bhubaneswar, India; 48 Panjab University, Chandigarh, India; 49 University of Delhi, Delhi, India; 50 Saha Institute of Nuclear Physics, Kolkata, India; 51 Bhabha Atomic Research Centre, Mumbai, India; 52 Tata Institute of Fundamental Research, Mumbai, India; 53 Indian Institute of Science Education and Research (IISER), Pune, India; 54 Institute for Research in Fundamental Sciences (IPM), Tehran, Iran; 55 University College Dublin, Dublin, Ireland; 56 INFN Sezione di Bari, Università di Bari, Politecnico di Bari, Bari, Italy; 57 INFN Sezione di Bologna, Università di Bologna, Bologna, Italy; 58 INFN Sezione di Catania, Università di Catania, Catania, Italy; 59 INFN Sezione di Firenze, Università di Firenze, Florence, Italy; 60 INFN Laboratori Nazionali di Frascati, Frascati, Italy; 61 INFN Sezione di Genova, Università di Genova, Genoa, Italy; 62 INFN Sezione di Milano-Bicocca, Università di Milano-Bicocca, Milan, Italy; 63 INFN Sezione di Napoli, Università di Napoli ‘Federico II’, Napoli, Italy, Università della Basilicata, Potenza, Italy, Università G. Marconi, Rome, Italy; 64 INFN Sezione di Padova, Università di Padova, Padova, Italy, Università di Trento, Trento, Italy; 65 INFN Sezione di Pavia, Università di Pavia, Pavia, Italy; 66 INFN Sezione di Perugia, Università di Perugia, Perugia, Italy; 67 INFN Sezione di Pisa, Università di Pisa, Scuola Normale Superiore di Pisa, Pisa, Italy; 68 INFN Sezione di Roma, Università di Roma, Rome, Italy; 69 INFN Sezione di Torino, Università di Torino, Torino, Italy, Università del Piemonte Orientale, Novara, Italy; 70 INFN Sezione di Trieste, Università di Trieste, Trieste, Italy; 71 Kangwon National University, Chunchon, Korea; 72 Kyungpook National University, Daegu, Korea; 73 Chonbuk National University, Jeonju, Korea; 74 Chonnam National University, Institute for Universe and Elementary Particles, Kwangju, Korea; 75 Korea University, Seoul, Korea; 76 Seoul National University, Seoul, Korea; 77 University of Seoul, Seoul, Korea; 78 Sungkyunkwan University, Suwon, Korea; 79 Vilnius University, Vilnius, Lithuania; 80 National Centre for Particle Physics, Universiti Malaya, Kuala Lumpur, Malaysia; 81 Centro de Investigacion y de Estudios Avanzados del IPN, Mexico City, Mexico; 82 Universidad Iberoamericana, Mexico City, Mexico; 83 Benemerita Universidad Autonoma de Puebla, Puebla, Mexico; 84 Universidad Autónoma de San Luis Potosí, San Luis Potosí, Mexico; 85 University of Auckland, Auckland, New Zealand; 86 University of Canterbury, Christchurch, New Zealand; 87 National Centre for Physics, Quaid-I-Azam University, Islamabad, Pakistan; 88 National Centre for Nuclear Research, Swierk, Poland; 89 Institute of Experimental Physics, Faculty of Physics, University of Warsaw, Warsaw, Poland; 90 Laboratório de Instrumentação e Física Experimental de Partículas, Lisboa, Portugal; 91 Joint Institute for Nuclear Research, Dubna, Russia; 92 Petersburg Nuclear Physics Institute, Gatchina, St. Petersburg, Russia; 93 Institute for Nuclear Research, Moscow, Russia; 94 Institute for Theoretical and Experimental Physics, Moscow, Russia; 95 National Research Nuclear University ‘Moscow Engineering Physics Institute’ (MEPhI), Moscow, Russia; 96 P.N. Lebedev Physical Institute, Moscow, Russia; 97 Skobeltsyn Institute of Nuclear Physics, Lomonosov Moscow State University, Moscow, Russia; 98 State Research Center of Russian Federation, Institute for High Energy Physics, Protvino, Russia; 99 Faculty of Physics and Vinca Institute of Nuclear Sciences, University of Belgrade, Belgrade, Serbia; 100 Centro de Investigaciones Energéticas Medioambientales y Tecnológicas (CIEMAT), Madrid, Spain; 101 Universidad Autónoma de Madrid, Madrid, Spain; 102 Universidad de Oviedo, Oviedo, Spain; 103 Instituto de Física de Cantabria (IFCA), CSIC-Universidad de Cantabria, Santander, Spain; 104 CERN, European Organization for Nuclear Research, Geneva, Switzerland; 105 Paul Scherrer Institut, Villigen, Switzerland; 106 Institute for Particle Physics ETH Zurich, Zurich, Switzerland; 107 Universität Zürich, Zurich, Switzerland; 108 National Central University, Chung-Li, Taiwan; 109 National Taiwan University (NTU), Taipei, Taiwan; 110 Faculty of Science, Department of Physics, Chulalongkorn University, Bangkok, Thailand; 111 Cukurova University, Adana, Turkey; 112 Physics Department, Middle East Technical University, Ankara, Turkey; 113 Bogazici University, Istanbul, Turkey; 114 Istanbul Technical University, Istanbul, Turkey; 115 Institute for Scintillation Materials of National Academy of Science of Ukraine, Kharkov, Ukraine; 116 National Scientific Center, Kharkov Institute of Physics and Technology, Kharkov, Ukraine; 117 University of Bristol, Bristol, UK; 118 Rutherford Appleton Laboratory, Didcot, UK; 119 Imperial College, London, UK; 120 Brunel University, Uxbridge, UK; 121 Baylor University, Waco, USA; 122 The University of Alabama, Tuscaloosa, USA; 123 Boston University, Boston, USA; 124 Brown University, Providence, USA; 125 University of California, Davis, Davis, USA; 126 University of California, Los Angeles, USA; 127 University of California, Riverside, Riverside, USA; 128 University of California, San Diego, La Jolla, USA; 129 University of California, Santa Barbara, Santa Barbara, USA; 130 California Institute of Technology, Pasadena, USA; 131 Carnegie Mellon University, Pittsburgh, USA; 132 University of Colorado Boulder, Boulder, USA; 133 Cornell University, Ithaca, USA; 134 Fermi National Accelerator Laboratory, Batavia, USA; 135 University of Florida, Gainesville, USA; 136 Florida International University, Miami, USA; 137 Florida State University, Tallahassee, USA; 138 Florida Institute of Technology, Melbourne, USA; 139 University of Illinois at Chicago (UIC), Chicago, USA; 140 The University of Iowa, Iowa City, USA; 141 Johns Hopkins University, Baltimore, USA; 142 The University of Kansas, Lawrence, USA; 143 Kansas State University, Manhattan, USA; 144 Lawrence Livermore National Laboratory, Livermore, USA; 145 University of Maryland, College Park, USA; 146 Massachusetts Institute of Technology, Cambridge, USA; 147 University of Minnesota, Minneapolis, USA; 148 University of Mississippi, Oxford, USA; 149 University of Nebraska-Lincoln, Lincoln, USA; 150 State University of New York at Buffalo, Buffalo, USA; 151 Northeastern University, Boston, USA; 152 Northwestern University, Evanston, USA; 153 University of Notre Dame, Notre Dame, USA; 154 The Ohio State University, Columbus, USA; 155 Princeton University, Princeton, USA; 156 University of Puerto Rico, Mayagüez, USA; 157 Purdue University, West Lafayette, USA; 158 Purdue University Calumet, Hammond, USA; 159 Rice University, Houston, USA; 160 University of Rochester, Rochester, USA; 161 Rutgers, The State University of New Jersey, Piscataway, USA; 162 University of Tennessee, Knoxville, USA; 163 Texas A&M University, College Station, USA; 164 Texas Tech University, Lubbock, USA; 165 Vanderbilt University, Nashville, USA; 166 University of Virginia, Charlottesville, USA; 167 Wayne State University, Detroit, USA; 168 University of Wisconsin-Madison, Madison, WI USA; 169 CERN, Geneva, Switzerland

## Abstract

A measurement of the decorrelation of azimuthal angles between the two jets with the largest transverse momenta is presented for seven regions of leading jet transverse momentum up to 2.2$\,\mathrm{TeV}$. The analysis is based on the proton-proton collision data collected with the CMS experiment at a centre-of-mass energy of 8$\,\mathrm{TeV}$ corresponding to an integrated luminosity of 19.7$\,\text {fb}^{-1}$. The dijet azimuthal decorrelation is caused by the radiation of additional jets and probes the dynamics of multijet production. The results are compared to fixed-order predictions of perturbative quantum chromodynamics (QCD), and to simulations using Monte Carlo event generators that include parton showers, hadronization, and multiparton interactions. Event generators with only two outgoing high transverse momentum partons fail to describe the measurement, even when supplemented with next-to-leading-order QCD corrections and parton showers. Much better agreement is achieved when at least three outgoing partons are complemented through either next-to-leading-order predictions or parton showers. This observation emphasizes the need to improve predictions for multijet production.

## Introduction

Hadronic jets with large transverse momenta ${p}_{\mathrm {T}}$ are produced in high-energy proton-proton collisions when two partons interact with high momentum transfer via the strong force. At leading order (LO) in perturbative quantum chromodynamics (pQCD), two final-state partons are produced back-to-back in the transverse plane. For this case, the azimuthal angular separation between the two leading ${p}_{\mathrm {T}}$ jets in the transverse plane, $\Delta \phi _\text {dijet} =|\phi _\text {jet1}-\phi _\text {jet2} |$, equals $\pi$. The nonperturbative effects of multiparton interactions or hadronization disturb this correlation only mildly, and $\Delta \phi _\text {dijet} \approx \pi$ still holds. However, the production of a third high-${p}_{\mathrm {T}}$ jet leads to a decorrelation in azimuthal angle. The smallest achievable value of $\Delta \phi _\text {dijet} =2\pi /3$ occurs in a symmetric star-shaped 3-jet configuration. Fixed-order calculations in pQCD for 3-jet production with up to four outgoing partons provide next-to-leading-order (NLO) predictions for the region of $2\pi /3 \le \Delta \phi _\text {dijet} < \pi$. If more than three jets are produced, the azimuthal angle between the two leading jets can approach zero, although very small angular separations are suppressed because of the finite jet sizes for a particular jet algorithm. The measurement of the dijet azimuthal angular decorrelation is an interesting tool to gain insight into multijet production processes without measuring jets beyond the leading two.

This paper reports the measurement of the normalized dijet differential cross section as a function of the dijet azimuthal angular separation,

1 $$\begin{aligned} \frac{1}{\sigma _\text {dijet}} \frac{{\mathrm{d}}\sigma _\text {dijet}}{{\mathrm{d}}\Delta \phi _\text {dijet}}, \end{aligned}$$

for seven regions of the leading jet ${p}_{\mathrm {T}}$, ${p}_{\mathrm {T}} ^{\text {max}}$, within a rapidity region of $|y |<2.5$. Experimental and theoretical uncertainties are reduced by normalizing the $\Delta \phi _\text {dijet}$ distribution to the total dijet cross section $\sigma _\text {dijet}$ within each region of ${p}_{\mathrm {T}} ^{\text {max}}$. For the first time, azimuthal angular separations $\Delta \phi _\text {dijet}$ over the full phase space from 0 to $\pi$ are covered. Comparisons are made to fixed-order predictions up to NLO for 3-jet production, and to NLO and LO dijet as well as to tree-level multijet production, each matched with parton showers and complemented with multiparton interactions and hadronization.

The measurement is performed using data collected during 2012 with the CMS experiment at the CERN LHC, corresponding to an integrated luminosity of 19.7$\,\text {fb}^{-1}$ of proton-proton collisions at $\sqrt{s}=8\,\mathrm{TeV}$. Previous measurements of dijet azimuthal decorrelation were reported by the D0 Collaboration in $\mathrm {p}\overline{{\mathrm{p}}}$ collisions at $\sqrt{s}=1.96\,\mathrm{TeV}$ at the Tevatron [1, 2], and by the CMS and ATLAS Collaborations in $\mathrm {p}\mathrm {p}$ collisions at $\sqrt{s}=7\,\mathrm{TeV}$ at the LHC [3, 4].

## The CMS detector

A detailed description of the CMS detector, together with a definition of the coordinate system used and the relevant kinematic variables, can be found in Ref. [5]. The central feature of the CMS detector is a superconducting solenoid, 13 m in length and 6 m in inner diameter, providing an axial magnetic field of 3.8 T. Within the field volume are a silicon pixel and strip tracker, a lead tungstate crystal electromagnetic calorimeter (ECAL) and a brass and scintillator hadron calorimeter (HCAL), each composed of a barrel and two endcap sections. Charged particle trajectories are measured by the tracker with full azimuthal coverage within pseudorapidities $|\eta |< 2.5$. The ECAL, which is equipped with a preshower detector in the endcaps, and the HCAL cover the region $|\eta |< 3$. In addition to the barrel and endcap detectors, CMS has extensive forward calorimetry, which extends the coverage up to $|\eta |< 5$. Finally, muons are measured up to $|\eta |< 2.4$ by gas-ionization detectors embedded in the steel flux-return yoke outside the solenoid.

## Event reconstruction and selection

This measurement uses data samples that were collected with single-jet high-level triggers (HLT) [6]. Four such single-jet HLTs were considered that require at least one jet in the event to have ${p}_{\mathrm {T}} > 140$, 200, 260, and $320 \,\mathrm{GeV}$, respectively. All triggers were prescaled during the 2012 run except the highest-threshold trigger. The integrated luminosity $\mathcal {L}$ for the four trigger samples is shown in Table 1. The trigger efficiency is estimated using triggers with lower ${p}_{\mathrm {T}}$ thresholds. Using these four jet-energy thresholds gives 100 % trigger efficiencies in the corresponding four momentum regions $200<{p}_{\mathrm {T}} ^{\text {max}} <300\,\mathrm{GeV}$, $300<{p}_{\mathrm {T}} ^{\text {max}} <400\,\mathrm{GeV}$, $400<{p}_{\mathrm {T}} ^{\text {max}} <500\,\mathrm{GeV}$, and ${p}_{\mathrm {T}} ^{\text {max}} >500\,\mathrm{GeV}$.

**Table 1:** The integrated luminosity for each trigger sample considered in this analysis

HLT ${p}_{\mathrm {T}}$ threshold ($\text {GeV}$ )	140	200	260	320
$\mathcal {L}$ ($\text {fb}^{-1}$)	0.06	0.26	1.06	19.7

Particles are reconstructed and identified using a particle-flow (PF) algorithm, which combines the information from the individual subdetectors [7, 8]. The four-vectors of particle candidates, reconstructed by the above technique, are used as input to the jet-clustering algorithm. Jets are reconstructed using the infrared- and collinear-safe anti-${k}_{\mathrm {T}}$ clustering algorithm with a distance parameter $R=0.7$ [9]. The clustering is performed with the FastJet package [10] using four-momentum summation.

The reconstructed jets require small additional energy corrections to account for various reconstruction inefficiencies in tracks and clusters in the PF algorithm. These jet energy corrections [11] are derived using (1) simulated events, generated with pythia 6.4.22 [12] with tune Z2* [13, 14] and processed through the CMS detector simulation based on Geant4 [15], and (2) measurements containing dijet, photon+jet, and Z+jet events. The jet energy corrections, which depend on the $\eta$ and ${p}_{\mathrm {T}}$ of the jet, are applied to the jet four-momentum vectors as multiplicative factors [16]. The overall factor is typically 1.2 or smaller, approximately uniform in $\eta$, and is 1.05 or smaller for jets having ${p}_{\mathrm {T}} > 100\,\mathrm{GeV}$. An offset correction is applied to take into account the extra energy clustered into jets from additional proton-proton interactions within the same or neighbouring bunch crossings (in-time and out-of-time pileup) [11]. Pileup effects are important only for jets with low ${p}_{\mathrm {T}}$ and become negligible for jets with ${p}_{\mathrm {T}} > 200 \,\mathrm{GeV}$. The current measurement is, therefore, insensitive to pileup effects on jet energy calibration.

Each event is required to have at least one vertex reconstructed offline [17] with a position along the beam line that is within 24 cm of the nominal interaction point. To suppress nonphysical jets, i.e. jets resulting from noise in the ECAL and/or HCAL calorimeters, stringent criteria [18] are applied for identifying jets: each jet should contain at least two particles, one of which is a charged hadron, and the jet energy fraction carried by neutral hadrons and photons should be less than 90 %. The efficiency for identifying physical jets using these criteria is greater than 99 %.

The two leading jets, which define $\Delta \phi _\text {dijet}$, are selected by considering all jets in the event with ${p}_{\mathrm {T}} > 100 \,\mathrm{GeV}$ and an absolute rapidity $|y |<5$. Events are selected in which the leading jet ${p}_{\mathrm {T}}$ exceeds 200$\,\mathrm{GeV}$ and the rapidities $y_1$ and $y_2$ of the two leading jets lie within the tracker coverage of $|y |<2.5$.

**Fig. 1:**
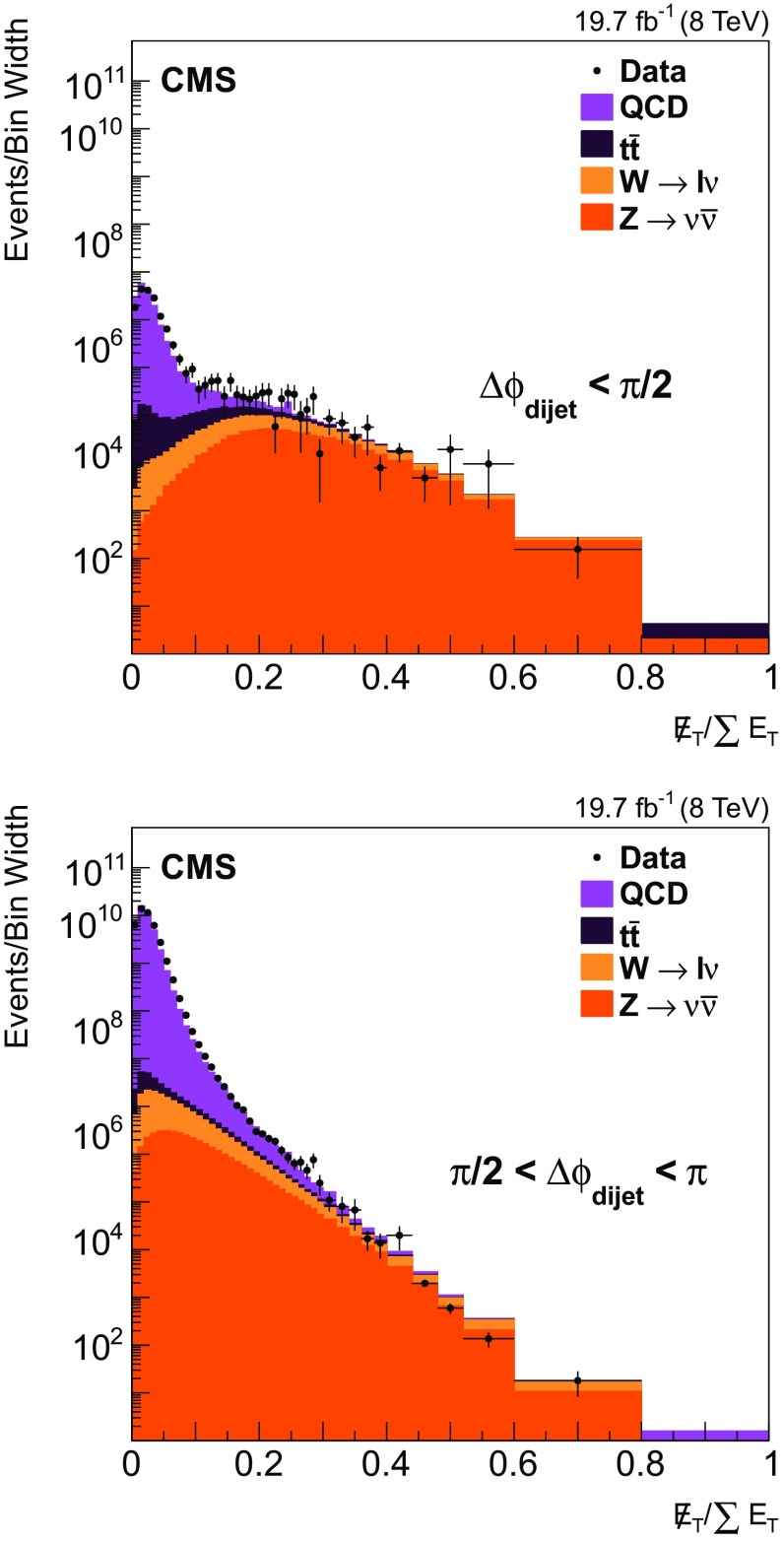
Distribution of $E_{\mathrm{T}}//\sum {{E}_{\mathrm {T}}}$ for data (*points*) in comparison with simulated jet production and other processes with large $E_{\mathrm{T}}/$ (*stacked*) separately for the two regions $\Delta \phi _\text {dijet} < \pi /2$ (*top*) and $\pi /2< \Delta \phi _\text {dijet} < \pi$ (*bottom*). The main contribution of events with large $E_{\mathrm{T}}/$ in the final state is caused by processes such as Z/W + jet(s) with ${\mathrm{Z}} \rightarrow \nu \overline{\nu }$ and $\mathrm {W}\rightarrow \ell \nu$

To reduce the background from ${\mathrm{t}}\overline{{\mathrm{t}}}$ and heavy vector boson production, the variable $E_{\mathrm{T}}//\sum {{E}_{\mathrm {T}}}$ is used. The sum of the transverse energies is $\sum {{E}_{\mathrm {T}}} = \sum _{i}E_{i}\sin \theta _{i}$, and the missing transverse energy $E_{\mathrm{T}}/ = \sqrt{ \left[ \sum _{i}\left( E_{i}\sin \theta _{i}\cos \phi _{i}\right) \right] ^2 + \left[ \sum _{i}\left( E_{i}\sin \theta _{i}\sin \phi _{i}\right) \right] ^2 }$, where $\theta$ is the polar angle and the sum runs over all PF candidates in the event. A noticeable fraction of high-${p}_{\mathrm {T}}$ jet events with large $E_{\mathrm{T}}/$ emerges from ${\mathrm{t}}\overline{{\mathrm{t}}}$ production with semileptonically decaying b quarks. In addition, Z/W+jet(s) events with Z decays to neutrinos and W decays into charged leptons with neutrinos have high $E_{\mathrm{T}}/$ values. The distributions of the variable $E_{\mathrm{T}}//\sum {{E}_{\mathrm {T}}}$ are shown in Fig. 1 for the two regions $\Delta \phi _\text {dijet} < \pi /2$ (top) and $\pi /2< \Delta \phi _\text {dijet} < \pi$ (bottom). The data (points) are compared to simulated events (stacked), using MadGraph 5.1.3.30 [19] matched to pythia6 [12] for event generation. Although some deviations of the simulation with respect to the data are visible in Fig. 1 (cf. Ref. [20]), the distributions allow a selection criterion to be optimized with respect to the ratio of signal over background. Events with $E_{\mathrm{T}}//\sum {{E}_{\mathrm {T}}} >0.1$ are rejected in both regions of $\Delta \phi _\text {dijet}$ considered in Fig. 1, which corresponds to about 0.7 % of the data sample. Negligible background fractions of $\approx$1 % and $\approx$0.1 % remain for the two regions $\Delta \phi _\text {dijet} < \pi /2$ and $\pi /2< \Delta \phi _\text {dijet} < \pi$, respectively.

## Measurement of the dijet cross section differential in $\Delta \phi _\text {dijet}$

The normalized dijet cross section differential in $\Delta \phi _\text {dijet}$ (Eq. 1) is corrected for detector smearing effects and unfolded to the level of stable (decay length $c\tau > 1~\mathrm{cm}$) final-state particles. In this way, a direct comparison of the measurement with corresponding results from other experiments and with QCD predictions can be made.

The unfolding method is based on the matrix inversion algorithm implemented in the software package RooUnfold [21]. Unfolding uses a response matrix that maps the distribution at particle-level onto the measured one. The response matrix is derived from a simulation that uses the true dijet cross section distribution from pythia6 with tune Z2* [13] as input, and introduces the smearing effects by taking into account the $\Delta \phi _\text {dijet}$ resolution. As a cross-check, the response matrix was filled from event samples that have been passed through a detector simulation. No significant difference was observed. The unfolded distributions differ from the raw distributions by 3–4 % for $\Delta \phi _\text {dijet} < \pi /2$ and by less than 3 % for $\pi /2< \Delta \phi _\text {dijet} < \pi$. A two-dimensional unfolding based on the iterative D’Agostini algorithm [22], which corrects for the smearing effects by taking into account both $\Delta \phi _\text {dijet}$ and ${p}_{\mathrm {T}}$ resolutions, gives almost identical results.

The main systematic uncertainties arise from the estimation of the jet energy scale (JES) calibration, the jet ${p}_{\mathrm {T}}$ resolution, and the unfolding correction. The JES uncertainty is estimated to be 1.0–2.5 % for PF jets, depending on the jet ${p}_{\mathrm {T}}$ and $\eta$ [11, 16, 23]. The resulting uncertainties in the normalized $\Delta \phi _\text {dijet}$ distributions range from 7 % at $\Delta \phi _\text {dijet} \approx 0$ via 3 % at $\pi /2$ to 1 % at $\pi$.

The jet ${p}_{\mathrm {T}}$ resolution is determined from a full detector simulation using events generated by pythia6 with tune Z2*, and is scaled by factors derived from data [11]. The effect of the jet ${p}_{\mathrm {T}}$ resolution uncertainty is estimated by varying it by one standard deviation up and down, and comparing the $\Delta \phi _\text {dijet}$ distributions before and after the changes. This results in a variation in the normalized $\Delta \phi _\text {dijet}$ distributions ranging from 5 % at $\Delta \phi _\text {dijet} \approx 0$ via 3 % at $\pi /2$ to 0.5 % at $\pi$.

The uncertainty in the unfolding correction factors is estimated by checking the dependence of the response matrix on the choice of the Monte Carlo (MC) generator. An alternative response matrix is built using the herwig++ 2.5.0 [24] event generator with the default tune of version 2.3. The observed effect is less than 1 %. An additional systematic uncertainty obtained by varying the $\Delta \phi _\text {dijet}$ resolution by ±10 % to determine the unfolding correction factors is estimated to be of the order of 1 %. This variation of the $\Delta \phi _\text {dijet}$ resolution by ±10 % is motivated by the observed difference between data and simulation in the $\Delta \phi _\text {dijet}$ resolution. A total systematic unfolding uncertainty of 1 % accounts for the choice of the MC generator in building the response matrix and the $\Delta \phi _\text {dijet}$ resolution.

**Fig. 2:**
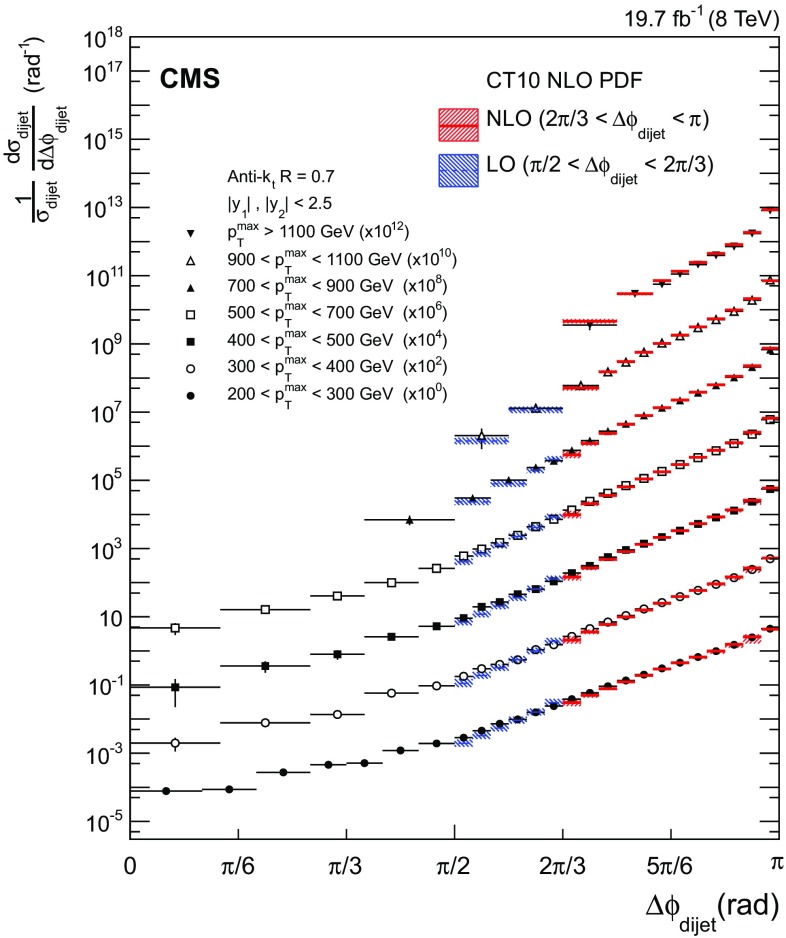
Normalized dijet cross section differential in $\Delta \phi _\text {dijet}$ for seven ${p}_{\mathrm {T}} ^{\text {max}}$ regions, scaled by multiplicative factors for presentation purposes. The *error bars* on the data points include statistical and systematic uncertainties. Overlaid on the data (*points*) are predictions from LO (*dashed line*; $\pi /2 \le \Delta \phi _\text {dijet} < 2\pi /3$) and NLO (*solid line*; $2\pi /3 \le \Delta \phi _\text {dijet} \le \pi$) calculations using the CT10 NLO PDF set. The PDF, $\alpha _S$, and scale uncertainties are added in quadrature to give the total theoretical uncertainty, which is indicated by the *downwards-diagonally* (LO) and *upwards-diagonally* (NLO) hatched regions around the *theory lines*

The unfolded dijet cross section differential in $\Delta \phi _\text {dijet}$ and normalized by the dijet cross section integrated over the entire phase space is shown in Fig. 2 for seven ${p}_{\mathrm {T}} ^{\text {max}}$ regions. Each region is scaled by a multiplicative factor for presentation purposes. The $\Delta \phi _\text {dijet}$ distributions are strongly peaked at $\pi$ and become steeper with increasing ${p}_{\mathrm {T}} ^{\text {max}}$. Overlaid on the data for $\Delta \phi _\text {dijet} > \pi /2$ are predictions from pQCD, presented in more detail in the next section, using parton distribution functions (PDF) of the CT10 PDF set.

## Comparison to theoretical predictions

### Predictions from fixed-order calculations in pQCD

The theoretical predictions for the normalized dijet cross section differential in $\Delta \phi _\text {dijet}$ are based on a 3-jet calculation at NLO. The correction of nonperturbative (NP) effects, which account for multiparton interactions (MPI) and hadronization, is studied using event samples simulated with the pythia6 (tune Z2*) and herwig++ (tune 2.3) event generators. Small NP effects are expected, since this measurement deals with a normalized distribution. These corrections are found to be of the order of 1 %, roughly at the limit of the accuracy of the MC simulations. Therefore NP corrections are considered to be negligible and are not applied.

The fixed-order calculations are performed using the NLOJet++ program version 4.1.3 [25, 26] within the framework of the fastNLO package version 2.3.1 [27]. The differential cross section is calculated for 3-jet production at NLO, i.e. up to terms of order $\alpha _S ^{4}$, with three or four partons in the final state. This calculation has LO precision in the region $\pi /2 \le \Delta \phi _\text {dijet} < 2\pi /3$ and NLO precision for $2\pi /3 \le \Delta \phi _\text {dijet} < \pi$. The bin including $\Delta \phi _\text {dijet} = \pi$ is computed from the NLO dijet cross section within this bin. For each region in ${p}_{\mathrm {T}} ^{\text {max}}$, the differential cross section is normalized to the dijet cross section calculated at LO for $\pi /2 \le \Delta \phi _\text {dijet} < 2\pi /3$ and at NLO, i.e. up to terms proportional to $\alpha _S ^{3}$, for $2\pi /3 \le \Delta \phi _\text {dijet} \le \pi$. The use of the LO dijet cross section for the normalization in the region $\pi /2 \le \Delta \phi _\text {dijet} < 2\pi /3$ leads to an improved description of the data and avoids artificially increased scale uncertainties as described in Refs. [28, 29]. Of course, this difference in normalization leads to a discontinuity proportional to $\sigma _\text {dijet}^\text {NLO}/\sigma _\text {dijet}^\text {LO}$ at $\Delta \phi _\text {dijet} = 2\pi /3$.

The number of quark flavours that are assumed to be massless is set to five, and the renormalization and factorization scales, $\mu _r$ and $\mu _f$, are chosen to be equal to ${p}_{\mathrm {T}} ^{\text {max}}$. The PDF sets with NLO evolutions used in the calculations are tabulated in Table 2. The ABM11 PDF set utilizes a fixed flavour number scheme, whereas the rest of the PDF sets use a variable flavour number scheme. The maximum number of flavours is denoted by $N_f$.

**Table 2:** The PDF sets used to compare the data with expectations, together with the corresponding maximum number of flavours $N_f$ and the default values of $\alpha _S(M_{{\mathrm{Z}}})$

Base set	Refs.	$N_f$	$\alpha _S(M_{{\mathrm{Z}}})$
ABM11	[30]	5	0.1180
CT10	[31]	${\le }$5	0.1180
HERAPDF1.5	[32]	${\le }$5	0.1176
MSTW2008	[33]	${\le }$5	0.1202
NNPDF21	[34]	${\le }$6	0.1190

**Fig. 3:**
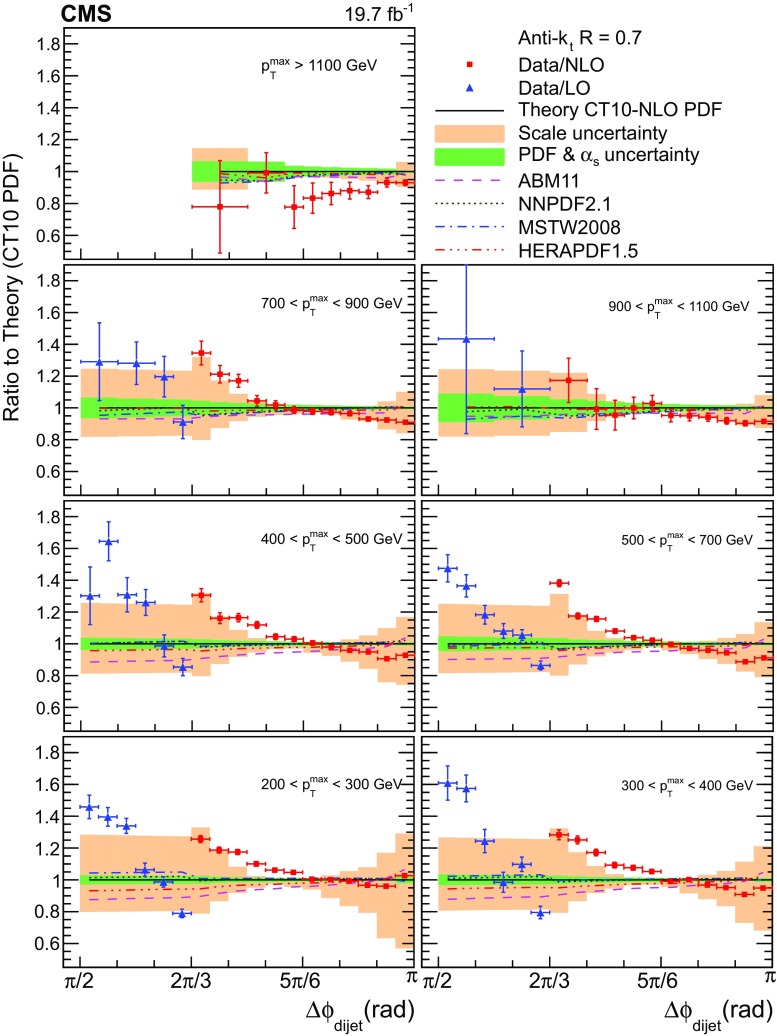
Ratios of the normalized dijet cross section differential in $\Delta \phi _\text {dijet}$ to LO (*triangles*) and NLO (*squares*) pQCD predictions using the CT10 PDF set at next-to-leading evolution order for all ${p}_{\mathrm {T}} ^{\text {max}}$ regions. The *error bars* on the data points represent the total experimental uncertainty, which is the quadratic sum of the statistical and systematic uncertainties. The uncertainties of the theoretical predictions are shown as *inner band* (PDF & $\alpha _S$) and *outer band* (*scales*). The predictions using various other PDF sets relative to CT10 are indicated with different line styles

The uncertainties due to the renormalization and factorization scales are evaluated by varying the default choice of $\mu _r = \mu _f = {p}_{\mathrm {T}} ^{\text {max}}$ between ${p}_{\mathrm {T}} ^{\text {max}}$/2 and 2${p}_{\mathrm {T}} ^{\text {max}}$, simultaneously in the differential cross section and in the total cross section, in the following six combinations: $(\mu _r/{p}_{\mathrm {T}} ^{\text {max}}, \mu _f/{p}_{\mathrm {T}} ^{\text {max}}) = (1/2,1/2)$, (1 / 2, 1), (1, 1 / 2), (1, 2), (2, 1), and (2, 2). The PDF uncertainties are evaluated according to the prescriptions for the CT10 PDF set in Ref. [35]. The CT10 PDF set employs the eigenvector method with upward and downward variations for each eigenvector. To evaluate the uncertainty due to the value of the strong coupling constant at 68 % confidence level, $\alpha _S(M_{{\mathrm{Z}}})$ is varied by ±0.001 as recommended in Ref. [36].

The results of fixed-order calculations with the CT10 PDF set are overlaid on the data for $\Delta \phi _\text {dijet} > \pi /2$ in Fig. 2. Figure 3 shows the ratio of the normalized dijet cross section differential in $\Delta \phi _\text {dijet}$ to theory calculated using the CT10 PDF set, together with the combined PDF and $\alpha _S$ uncertainty (inner band), and the scale uncertainty (outer band). Also shown are the ratios of theory derived with the alternative PDF sets ABM11 (dashed line), HERAPDF1.5 (dashed–three-dotted line), MSTW2008 (dashed-dotted line), and NNPDF2.1 (dotted line) compared to the prediction with the CT10 PDFs.

The fixed-order calculations agree with the data for azimuthal angular separations larger than $5\pi /6$ except for the highest ${p}_{\mathrm {T}} ^{\text {max}}$ region, where they exceed the data. For smaller $\Delta \phi _\text {dijet}$ values between $2\pi /3$ and $5\pi /6$, in particular where the estimate of the theoretical uncertainties becomes small, systematic discrepancies are exhibited that diminish with increasing ${p}_{\mathrm {T}} ^{\text {max}}$. In the 4-jet LO region with $\Delta \phi _\text {dijet} < 2\pi /3$, the pattern of increasing deviations towards smaller $\Delta \phi _\text {dijet}$ and decreasing deviations towards larger ${p}_{\mathrm {T}} ^{\text {max}}$ is repeated, but with less significance because of the larger scale uncertainty. Similar observations were made in the previous CMS measurement [3], which exhibited larger discrepancies in the 4-jet region due to the normalization to the NLO dijet cross section instead of a LO one.

### Predictions from fixed-order calculations matched to parton shower simulations

The pythia6 [12], pythia8 [37], and herwig++ [24] event generators complement LO dijet matrix elements with parton showers to simulate higher-order processes. Both pythia versions, pythia6 with the Z2* tune [13] and pythia8 with the CUETM1 tune [14], employ ${p}_{\mathrm {T}}$-ordered parton showers [38, 39], while herwig++ with the default tune of version 2.3 uses a coherent-branching algorithm with angular ordering of the showers [40].

**Fig. 4:**
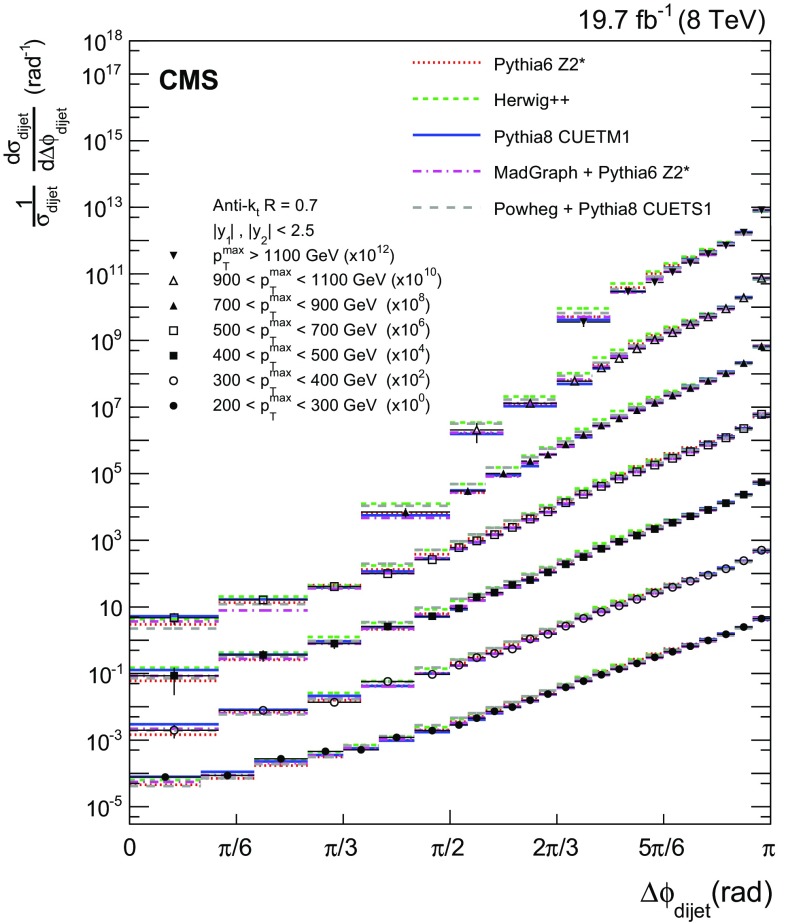
Normalized dijet cross section differential in $\Delta \phi _\text {dijet}$ for seven ${p}_{\mathrm {T}} ^{\text {max}}$ regions, scaled by multiplicative factors for presentation purposes. The *error bars* on the data points include statistical and systematic uncertainties. Overlaid on the data are predictions from the pythia6, herwig++, pythia8, MadGraph + pythia6, and powheg + pythia8 event generators

The MadGraph program version 5.1.5.7 [19] supplies the results of LO matrix element calculations with two to four outgoing partons that can be matched to the implementations of parton showers, hadronization, and MPI of the event generators. In this analysis, it is interfaced with pythia6 with tune Z2* using the MLM matching procedure [41] to avoid any double counting between tree-level and parton shower generated parton configurations.

The powheg framework [42–44] provides an NLO dijet calculation [45] that can also be matched via the parton showers to event generators. Here, powheg is used with the CT10NLO PDF set and is interfaced to pythia8 with the CUET [14] tune, which employs the LO CTEQ6L1 [35] PDF set. Predictions with parton showers matched to a NLO 3-jet calculation using powheg [46] or MadGraph5_aMC@NLO [47] would be even more relevant for a multijet topology. They could not, however, be included within the timescale of this analysis. Approaching azimuthal angular separations close to $\pi$, it might also be interesting to compare to predictions employing the technique of ${p}_{\mathrm {T}}$ resummation [48].

**Fig. 5:**
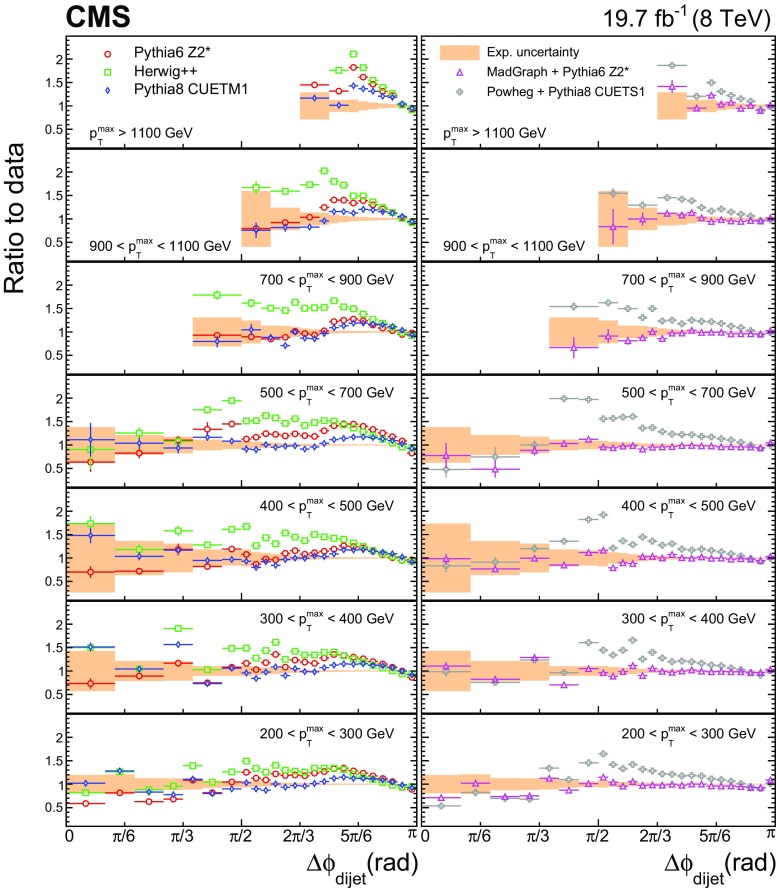
Ratios of pythia6, herwig++, pythia8, MadGraph + pythia6, and powheg + pythia8 predictions to the normalized dijet cross section differential in $\Delta \phi _\text {dijet}$, for all ${p}_{\mathrm {T}} ^{\text {max}}$ regions. The *solid band* indicates the total experimental uncertainty and the *error bars* on the MC points represent the statistical uncertainties of the simulated data

In Fig. 4 the normalized dijet cross section differential in $\Delta \phi _\text {dijet}$ is compared to the predictions from fixed-order calculations supplemented with parton showers, hadronization, and MPI. The error bars on the data points represent the total experimental uncertainty, which is the quadratic sum of the statistical and systematic uncertainties. Figure 5 shows the ratios of these predictions to the normalized dijet cross section differential in $\Delta \phi _\text {dijet}$, for the seven ${p}_{\mathrm {T}} ^{\text {max}}$ regions. The solid band indicates the total experimental uncertainty and the error bars on the MC points represent the statistical uncertainties in the simulated data.

Among the LO dijet event generators pythia6, pythia8, and herwig++, pythia8 exhibits the smallest deviations from the measurements. pythia6 and herwig++ systematically overshoot the data, particular around $\Delta \phi _\text {dijet} = 5\pi /6$. The best description of the measurement is given by the tree-level multiparton event generator MadGraph interfaced with pythia6 for showering, hadronization, and MPI. The powheg generator (here used only in the NLO dijet mode) matched to pythia8 shows deviations from the data similar to the LO dijet event generators.

## Summary

A measurement is presented of the normalized dijet cross section differential in the azimuthal angular separation $\Delta \phi _\text {dijet}$ of the two jets leading in ${p}_{\mathrm {T}}$ for seven regions in the leading-jet transverse momentum ${p}_{\mathrm {T}} ^{\text {max}}$. The data set of pp collisions at 8$\,\mathrm{TeV}$ centre-of-mass energy collected in 2012 by the CMS experiment and corresponding to an integrated luminosity of 19.7$\,\text {fb}^{-1}$ is analysed.

The measured distributions in $\Delta \phi _\text {dijet}$ are compared to calculations in perturbative QCD for 3-jet production with up to four outgoing partons that provide NLO predictions for the range of $2\pi /3 \le \Delta \phi _\text {dijet} < \pi$ and LO predictions for $\pi /2 \le \Delta \phi _\text {dijet} < 2\pi /3$. The NLO predictions describe the data down to values of $\Delta \phi _\text {dijet} \approx 5\pi /6$, but deviate increasingly when approaching the 4-jet region, starting at $\Delta \phi _\text {dijet} = 2\pi /3$, particularly at low ${p}_{\mathrm {T}} ^{\text {max}}$. The pattern of increasing deviations towards smaller $\Delta \phi _\text {dijet}$ and decreasing deviations towards larger ${p}_{\mathrm {T}} ^{\text {max}}$ is repeated in the 4-jet LO region with $\Delta \phi _\text {dijet} < 2\pi /3$, but with less significance because of the larger scale uncertainty.

In a comparison of the normalized $\Delta \phi _\text {dijet}$ distributions to the LO dijet event generators pythia6, pythia8, and herwig++, pythia8 gives the best agreement. pythia6 and herwig++ systematically overshoot the data, particularly for $\Delta \phi _\text {dijet} \approx 5\pi /6$. A good overall description of the measurement is provided by the tree-level multijet event generator MadGraph in combination with pythia6 for showering, hadronization, and multiparton interactions. The dijet NLO calculations from powheg matched to pythia8 exhibit deviations similar to the LO dijet event generators. Improved multijet predictions can be expected from 3-jet NLO calculations matched to parton showers like from powheg or MadGraph5_aMC@NLO.

Similar observations were reported previously by CMS [3] and ATLAS [4], but with less significance because of the smaller data sets. The extension to $\Delta \phi _\text {dijet}$ values below $\pi /2$, the improved LO description in the 4-jet region $\pi /2 \le \Delta \phi _\text {dijet} < 2\pi /3$, and the comparison to dijet NLO calculations matched to parton showers are new results of the present analysis.
